# Applying the WHO ICF Framework to the Outcome Measures Used in the Evaluation of Long-Term Clinical Outcomes in Coronavirus Outbreaks

**DOI:** 10.3390/ijerph17186476

**Published:** 2020-09-05

**Authors:** Kajal Patel, Sofia Straudi, Ng Yee Sien, Nora Fayed, John L. Melvin, Manoj Sivan

**Affiliations:** 1 School of Medicine, University of Manchester, Manchester M13 9PG, UK; kj.patel1020@gmail.com; 2 Division of Neurosciences and Experimental Psychology, University of Manchester, Manchester M13 9PL, UK; 3 Neuroscience and Rehabilitation Department, Ferrara University Hospital, 44100 Ferrara, Italy; s.straudi@ospfe.it; 4 Department of Rehabilitation Medicine, Singapore General Hospital, Outram Road, Singapore 169608, Singapore; ng.yee.sien@singhealth.com.sg; 5 School of Rehabilitation Therapy, Queen’s University, Louise D. Acton Building, 31 George Street, Kingston, ON K7L 3N6, Canada; nf31@queensu.ca; 6 Department of Rehabilitation Medicine, Sidney Kimmel Medical College, Thomas Jefferson University, Philadelphia, PA 19144, USA; honpres@hotmail.com; 7 Academic Department of Rehabilitation Medicine, University of Leeds and Leeds Teaching Hospitals NHS Trust, Leeds LS2 9JT, UK

**Keywords:** COVID-19, SARS, MERS, outcome measures, follow-up studies, prevalence, lung function, exercise tolerance, mental health, quality of life

## Abstract

(1) Objective: The World Health Organization’s (WHO) International Classification of Functioning, Disability and Health (ICF) classification is a unified framework for the description of health and health-related states. This study aimed to use the ICF framework to classify outcome measures used in follow-up studies of coronavirus outbreaks and make recommendations for future studies. (2) Methods: EMBASE, MEDLINE, CINAHL and PsycINFO were systematically searched for original studies assessing clinical outcomes in adult survivors of severe acute respiratory distress syndrome (SARS), middle east respiratory syndrome (MERS) and coronavirus disease-19 (COVID-19) after hospital discharge. Individual items of the identified outcome measures were linked to ICF second-level and third-level categories using ICF linking rules and categorized according to an ICF component. (3) Results: In total, 33 outcome measures were identified from 36 studies. Commonly used (a) ICF body function measures were Pulmonary Function Tests (PFT), Impact of event scale (IES-R) and Hospital Anxiety and Depression Scale (HADS); (b) ICF activity was 6-Minute Walking Distance (6MWD); (c) ICF participation measures included Short Form-36 (SF-36) and St George’s Respiratory Questionnaire (SGRQ). ICF environmental factors and personal factors were rarely measured. (4) Conclusions: We recommend future COVID-19 follow-up studies to use the ICF framework to select a combination of outcome measures that capture all the components for a better understanding of the impact on survivors and planning interventions to maximize functional return.

## 1. Introduction

Coronavirus disease 2019 (COVID-19) has posed a major challenge to healthcare systems worldwide, with over 6 million confirmed cases and 350,000 deaths reported as of May 2020 [1]. Whilst current efforts have been duly focused on reducing mortality, issues regarding potential long-term complications in COVID-19 survivors are starting to emerge [2,3,4]. In the previous coronavirus outbreaks of severe acute respiratory syndrome (SARS) and Middle East respiratory syndrome (MERS), such long-term sequelae spanned across the physical, psychological and social domains of health [5,6]. Early reports of COVID-19 follow-up studies are suggestive of prevalence of similar health problems in COVID-19 survivors as those reported in previous outbreaks of SARS and MERS [7,8]. Considering the multi-systemic impact of COVID-19, follow-up studies across all these domains of health are required in order to truly understand these individuals’ experiences and support their return to societal roles.

Post-acute early rehabilitation of coronavirus survivors would foremost involve recovery of body structure and function. Impairment of respiratory function [9,10,11], exercise tolerance [12] and neuromuscular functions [13] have been reported to be present in coronavirus survivors beyond 3 months post-infection, with improvement to some degree in many of these individuals. However, improvement in these impairments of body structure and function may not necessarily translate into recovery from disability and role limitation [14,15]. For instance, during the SARS outbreak in 2002, even though most patients had good recovery from their physical illness, their quality of life was still lower than healthy individuals and as many as 17% of coronavirus survivors had not returned to work 1-year post-discharge [11]. Furthermore, evaluation of impairment without the knowledge of personal factors and help available to them in their environments can be futile as barriers in these contextual factors need to be overcome to facilitate participation of the individual in society.

In order to assess survivors’ long-term health comprehensively and provide rehabilitation, all aspects of health that influence recovery, including organ impairments, functional limitations and personal circumstances, should be assessed. The World Health Organization’s International Classification of Functioning, Disability and Health (WHO ICF) model put forward in 2001 provides a coherent view of different aspects of health from biological, individual and social perspectives [16,17]. This framework has been extensively researched and validated for describing health state [17], epidemiology [18] and public health [18], classifying outcome measures [15,19] and planning interventions [16,20]. The interplay between these factors in COVID-19 survivors is important to recognize in order to characterize their multi-systemic problems and disability and improve “functioning” by targeted interventions.

The aim of this systematic review is to identify outcome measures which have been used in follow-up studies in the coronavirus outbreaks, including SARS in 2002 and MERS in 2012 [21], and to classify them using the ICF model. This will allow an understanding of whether all aspects of the health condition have been captured by these studies and highlight any gaps that need to be considered when selecting outcome measures for future studies.

## 2. Methods

This systematic review was conducted in two stages.

### 2.1. Stage 1. Identification of Long-Term Follow-Up Studies in Survivors of Previous Coronavirus Outbreaks

A comprehensive search of 4 databases, MEDLINE (1946 to Week 1 May 2020), EMBASE (1974 to 8th May 2020), CINAHL Plus (1937 to Week 1 May 2020) and PsycINFO (1806 to Week 1 May 2020), was performed. The search strategy used was [(Coronavirus OR Coronavirus Infections OR COVID OR SARS virus OR Severe acute respiratory syndrome OR MERS OR Middle east respiratory syndrome) AND (Follow-up OR Follow-up studies OR Prevalence)]. Terms were entered as MeSH terms where available for each database; otherwise, these were searched as keywords in the title, abstract and subject headings.

The searches were first screened using the abstract and then using the full text by two independent authors based on the inclusion and exclusion criteria below. Original studies involving adults with confirmed diagnosis of coronavirus infection who were followed up for any period post-discharge were included. Further inclusion was limited to studies that used at least one clinical outcome measure during the follow-up (e.g., studies investigating mortality only were excluded). Reviews, case reports and editorial reports were excluded. Information regarding the authors, study year and outcome measures used in all the included studies was extracted into standardized tables. Extraction was undertaken by KP and MS independently and then compared for accuracy.

### 2.2. Stage 2. Classification of Outcome Measures According to the Five Main ICF Components

ICF linking rules were used in this study. These rules have been developed specifically to link the content of each outcome measure to the ICF framework [22]. The rules suggest that meaningful concepts within the items of outcome measures should be identified and then linked to the most precise ICF category. Meaningful “concepts” are those that describe health condition, person, functional activity or any of the environmental factors. For example, the measure IES-R scale (Impact of Event Scale–Revised) has two items, “I had trouble staying asleep” and “I felt angry”, the concepts extracted from these items were sleep and anger. Sleep was linked to the second level ICF category, “b134-sleep function”, and anger was linked to the third level ICF category, “b1522-anger”. Meaningful concepts referring to “quality of life” are assigned to the “not definable—quality of life” category. If a meaningful concept is not contained in the ICF and is clearly a personal factor, it is assigned “personal factor”. If a meaningful concept is not contained in ICF and is not a personal factor, it is assigned “not covered”. If the meaningful concept refers to a diagnosis or a health condition, it is assigned “health condition” [22]. Based on the above rules, meaningful concepts were extracted from the items of the identified measures and linked to relevant ICF categories. Finally, each outcome measure was then classified to belong to the ICF component that covered the majority of its constituent questions. For example, the items of GHQ-12 were linked to the two ICF components, “body function and structures” and “activities”, with the majority of questions linking to the ICF “body function and structures” component. Hence, this outcome measure was classified as belonging to (or representing) ICF “body function and structures”. This stage was undertaken by authors KP and MS and cross-checked for consensus by NYS and NF.

Based on the spread of the these meaningful concepts, each outcome measure was classified into one of the main individual ICF components [22], defined as follows:

- Body structure and body function: refers to anatomical structure or physiological function such as those required for cognition, cardiovascular function, motor functions, pain or emotion.
- Activities: refers to the execution of tasks at an individual level.
- Participation: refers to the individual’s involvement in everyday life situations.
- Environmental factors: refer to physical, social and attitudinal factors in the person’s life and society which hinder or facilitate the functioning of the individual.
- Personal factors: refers to characteristics that are unique to each individual such as age, gender, ethnicity, personality, resilience or experiences.

This stage was undertaken independently by all authors and cross-checked for consensus.

## 3. Results

### 3.1. Stage 1. Study Selection

**Figure 1:**
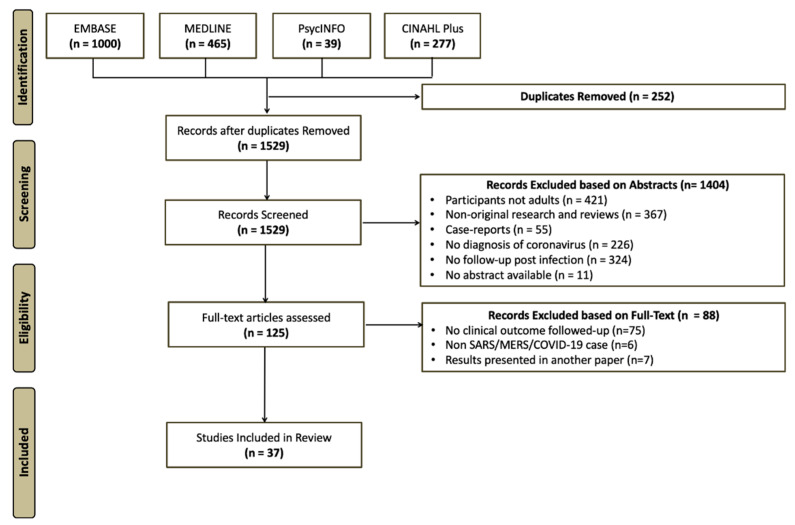
PRISMA flowchart for the literature search.

The search of four databases yielded 1528 studies. Out of these, 36 studies were finally included in this review. The reasons for exclusion of studies at each stage are reported in Figure 1.

### 3.2. Stage 2. ICF Outcome Measures

Table 1 and Table 2 summarizes the outcome measures used by the follow-up studies in coronavirus survivors included in this review. A total of 33 outcome measures were identified which measured several physical, psychological and quality of life outcomes. The commonly used outcome measures included Pulmonary Function Tests (PFT; 20 studies), Impact of Event Scale (IES-R; seven studies), Short-Form 36 (SF-36; six studies), 6-Minute Walking Distance (6MWD; five studies), Hospital Anxiety and Depression Scale (HADS; three studies) and St George’s Respiratory Questionnaire (SQRG; three studies).

**Figure 2:**
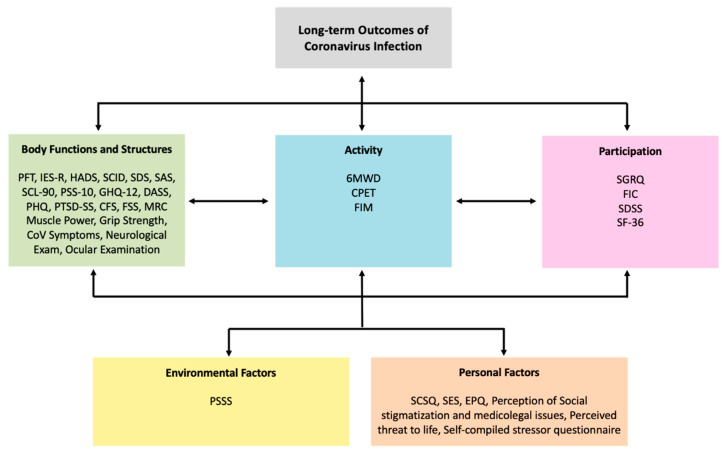
International Classification of Functioning, Disability and Health (ICF) categorization of outcome measures used in long-term follow-up studies of coronavirus survivors.

The mapping of meaningful concepts to the ICF categories is shown in Table 3. Based on how the units mapped, the authors decided on which ICF component each outcome measure ultimately represents. Figure 2 portrays the classification of outcome measures based on ICF components. In total, 19 outcome measures measured ICF body structure and function, three outcome measures measured activity, four outcome measures measured participation, one outcome measure measured environmental factors and seven outcome measures measured personal factors (see Table 1).

## 4. Discussion

Considering the widespread prevalence of COVID-19, the long-term complications of coronavirus infection in survivors will increase healthcare utilization significantly in the coming months. Previous outbreaks have shown that there is a broad spectrum of long-term complications of coronavirus infection which spans across all of the ICF components, with prevalence of respiratory compromise, exercise intolerance, psychological distress and reduced quality of life being considerably high several months following infection [5]. Deficits in different components do not necessarily translate into each other and there is no causal relationship between them [15]. The return of COVID-19 survivors to pre-infection levels of activity and participation in society are influenced not only by the degree of recovery from impairments in body structure and function but also by their personal and environmental factors, which hinder or facilitate their return to previous societal roles [54]. Therefore, it is essential that all future follow-up studies looking at COVID-19 survivors measure all aspects of the ICF framework. This review assimilates the outcome measures used in all the follow-up studies conducted during the present and past coronavirus outbreaks and classifies them according the ICF categories in order to provide a conceptual framework for the selection of outcome measures for future COVID-19 follow-up studies.

All studies included in this review reported outcomes in terms of body structure and function. The most commonly reported impairment in coronavirus survivors was respiratory compromise [9,10,11,12,34,35,36,39,42,43,44,48,55], with mainly restrictive patterns of lung function abnormality on pulmonary function testing. Neuromuscular impairment of muscle power and sensory function, particularly in those admitted to intensive care units, have also been reported based on neurological examination, MRC muscle power and grip strength measurements [13]. Long-term fatigue has also been measured using FSS and CFQ and found to be prevalent at 6 months post-discharge [24,27].

Alongside physical impairment, psychological impairments have also been widely elaborated by several studies. Prevalence of PTSD [23,24,26,27,29,38], depression [23,24,26,27] and anxiety [23,26] have been found to be particularly high in this cohort of patients. These mental health outcomes have been measured using a variety of scales. Outcome measures which were particularly useful in measuring impairment in these individuals were IES-R, PHQ-9 and GHQ-12 as these were able to capture the impact of psychological issues on a range of functions such as sleep, concentration, appetite and energy. We believe that at least two outcome measures should be used to assess impairment in body structure and function to address the physical and psychological impairment separately, such as a combination of lung function test and a PTSD outcome measure.

Limitations in activity have been measured through 6-min walking distance [9,12,31,45] and cardiopulmonary testing in the included studies [34]. Most of the studies report these to be reduced following discharge, with gradual improvement at 6 months post-discharge. Participation has been measured using SF-36, SGRQ and SDSS. These reflect several domains of self-care, domestic life, interpersonal relationships, mobility, work and social life. Quality of life has been reported to be considerably reduced in coronavirus survivors [11,29,31,42,56]. Despite having extensive impact on the overall wellbeing of an individual, these tools have been measured by only a minority of studies and should be measured consistently across all future follow-up studies in COVID-19 survivors. We recommend at least one functional measure of activity, such as walking distance, and one to capture participation and quality of life, such as SF-36, for future studies.

Environmental factors have not been explored adequately by the included studies. Only one study measured these through the perceived social support scale [28]. None of the studies provided or reported information regarding pulmonary rehabilitation, pharmacological interventions or psychological support. As these interventions are also considered as environmental factors which could facilitate the recovery of these impairments in survivors, reporting of such factors in COVID-19 patients is also important. Attitudes of family and society members have also not been explored. Some of the measures which have been used to measure environmental factors in other areas of health have been the Craig Hospital Inventory of Environmental Factors (CHIEF), Environmental Factors Item Bank (EFIB), Facilitators and Barrier’s Survey/Mobility (FABS/M) and Home and Community Environment Instrument (HACE). Future studies must aim to capture these factors along with family and carer support available in their chosen environments. Funding for rehabilitation will also play an important role in recovery for COVID-19 patients [57].

Personal factors which may play a role in recovery, such as coping styles, self-esteem, social stigma and personality, have been measured using self-constructed scales by some studies [24]. Some of the validated tools which have been used to measure such personal factors in other areas of health are the Connor Davidson resilience scale [58] and Kessler 6 psychological distress scale [59]. Inclusion of these measures could enable us to explore this domain further.

Through the use of the ICF framework, it is evident that, whilst impairments in body structure and function and restrictions in activity and participation have been measured extensively using standardized outcome measures, personal and environmental factors have only been measured in a small number of studies. The measurement of these contextual factors using standardized measures is essential as they have a major role to play in these individuals’ health and return to function.

Figure 2 describes our ICF framework approach for selecting outcome measures for future studies looking at long-term outcomes after COVID-19 illness. Apart from the measures suggested in this framework, other outcomes which could be used to measure environmental and personal factors are the Connor Davidson resilience scale, Kessler 6 psychological distress scale, Craig Hospital Inventory of Environmental Factors (CHIEF), Environmental Factors Item Bank (EFIB), Facilitators and Barrier’s Survey/Mobility (FABS/M) and Home and Community Environment Instrument (HACE) [60]. We propose that at least five different outcome measures spanning across all five ICF components need to be used in future follow-up studies.

It might be useful wherever possible for researchers to try to use the same outcome measures that were used in previous studies as this allows comparability and pooling of results. We however acknowledge that this might not be possible when there is a compelling case to use a measure that serves the purpose of the study better. For example, EQ5D is better suited to capture the health economics of the impact of the COVID-19 outbreak and researchers might opt to choose this over SF-36, which was predominantly used as the quality of life measure in previous studies.

The aim of this review was merely to categorize the currently available outcome measures into the ICF domains so that future researchers could pick an outcome measure corresponding to each category in order to capture the entire breadth of the health condition. We do not intend to make recommendations regarding specific outcome measures under each ICF domain that the reader should use. This would require further exploration of the psychometric properties of each measure, which is outside the scope of this paper. We only aim to provide a framework that one should keep in mind when choosing the measures rather than providing recommendations on which specific measure to use.

In the 36 studies included in this review, there were 33 different outcome measures used at follow-up. This makes comparison of results from studies using different measures difficult. In order to be able to do so, these diverse measures need to be converted into a common framework. This would most logically be done using the ICF, as it is the most commonly recognized international language of functioning. Thus, categorizing the measures into such a framework would be useful for researchers as it would identify the ICF categories that these authors found useful for their studies and inform their choice for future studies.

The main limitation of this review is that we have only described the outcome measures which have been used in the follow-up studies so far. This does not mean that outcome measures not used in these included studies are not suitable for use in future studies. Moreover, our search strategy was not designed to look for rehabilitation studies in coronavirus survivors. If these had been included in the review, then the set of outcome measures included in this review might have been slightly different. However, the aim of this review was to provide an ICF-based framework for the selection of outcome measures which researchers and clinicians are recommended to use. They need not necessarily use the same outcome measures as previous studies. For example, some researchers might prefer to use EQ5D instead of SF-36 for capturing quality of life. The rehabilitation community will be working hard in the next few years to help COVID-19 survivors achieve the best possible outcomes. The selection of outcome measures must be an essential first step rather than an afterthought in this process.

## 5. Conclusions

In conclusion, we are proposing an ICF-based framework to assist researchers in selecting outcome measures for future follow-up studies of COVID-19 survivors. This review highlighted that most studies so far placed greater emphasis on measuring body function impairments, limitations in activities and restrictions in participation. ICF personal and environmental factors were not as comprehensively covered and need to be included in COVID-19 follow-up studies. The individual ICF components are not linearly related and therefore a combination of outcome measures that capture all the components is recommended for a better understanding of the impact on survivors and planning interventions to maximize functional return.

## Figures and Tables

**Table 1:** Outcome measure abbreviations.

Abbreviation	Outcome Measure
PFT	Pulmonary Function Test
IES-R	Impact of Event Scale–Revised
HADS	Hospital Anxiety and Depression Scale
SCID	Structures Clinical Interview for DSM Disorders
SDS	Self-rating Depression Scale
SAS	Self-rating Anxiety Scale
SCL-90	Symptom Checklist 90
PSS-10	Perceived Stress Scale
GHQ-12	General Health Questionnaire 12
DASS	Depression Anxiety Stress Scale
PHQ	Patient Health Questionnaire 9
PTSD-SS	PTSD Self-rating Scale
CFS	Chalder Fatigue Scale
FSS	Fatigue Severity Scale
MRC Muscle Power	Medical Research Council Muscle Power
NMS Exam	Neuromuscular Examination
6MWD	6-Minute Walking Distance
CPET	Cardiopulmonary Exercise Testing
FIM	Functional Independence Measure
SGRQ	St George’s Respiratory Questionnaire
FIC	Functional Impairment Checklist
SDSS	Social Disability Screening Schedule
SF-36	Short-Form 36
PSSS	Perceived Social Support Scale
SCSQ	Simple Coping Style Questionnaire
SES	Self-Esteem Scale
EPQ	Eysenck Personality Questionnaire

**Table 2:** Outcome measures used in follow-up studies of coronavirus survivors.

Studies	Outcome Measures Used in Follow-Up Studies of Coronavirus Survivors																														
	PFT	IES-R	HADS	SCID	SDS	SAS	SCL-90	PSS-10	GHQ-12	DASS	PHQ-9	PTSD-SS	CFS	FSS	MRC Power	Grip Strength	Symptoms	NMS Exam	Ocular Exam	6MWD	CPET	FIM	SF-36	SGRQ	FIC	SDSS	PSSS	SCSQ	SES	EPQ	Others
**Mak et al. (2009)** [23]		+	+	+																			+								
**Lam et al. (2009)** [24]		+	+	+									+																		Perception of social stigmatization and medicolegal issues
**Wu et al. (2005)** [25]		+	+																												5-point scale measuring perceived threat to life
**Lee et al. (2007)** [26]		+						+	+	+																					
**Lee et al. (2019)** [27]		+									+			+																	
**Zhang et al. (2005)** [28]		+																									+	+	+	+	Self-compiled stressor questionnaire
**Hong et al. (2009)** [29]		+			+	+	+																+			+					
**Liu et al. (2020)** [30]	+				+	+														+		+	+								
**Lam et al. (2006)** [31]																+				+			+	+	+						
**Peng et al. (2003)** [18]	+																														
**Xie et al. (2005)** [32]	+																														
**HE et al. (2005)** [33]	+																														
**Ong et al. (2004)** [34]	+																				+										
**Ng et al. (2005)** [35]	+																														
**Zheng et al. (2005)** [36]	+																														
**Hsu et al. (2004)** [37]	+																+														
**Gao et al. (2006)** [38]												+																			
**Zheng-Yu et al. (2003)** [39]	+																														
**Yun et al. (2003)** [40]	+																														
**Liu et al. (2007)** [41]	+																														
**Ong et al. (2005)** [42]	+																							+							
**Zhang et al. (2020)** [43]	+																														
**Tsai et al. (2004)** [13]															+				+												
**Chen et al. (2006)** [10]																	+														
**Hui et al. (2005)** [9]	+																			+			+								
**Wong et al. (2004)** [44]	+																														
**Li et al. (2006)** [45]	+																			+			+								
**Park et al. (2018)** [12]	+																			+											
**Chiang et al. (2004)** [46]	+																														
**Yin et al. (2005)** [47]	+																														
**Wu et al. (2016)** [48]	+																														
**Tansey et al. (2007)** [11]	+																+			+				+							
**Avendano et al. (2003)** [49]																	+														
**Isakbaeva et al. (2004)** [50]																	+														
**Klopfenstein et al. (2020)** [51]																	+														
**Hopkins et al. (2020)** [52]																	+														
**Yuen et al. (2004)** [53]																		+													

“+” means that this outcome measure was investigated by the respective study.

**Table 3:** Mapping of outcome measure themes to ICF category codes.

ICF Code	Assessment	Body Functions and Structure																			Activity			Participation				Environment	Personal Factors *				
		PFT	IES-R	HADS	SCID	SDS	SAS	SCL-90	PSS-10	GHQ-12	DASS	PHQ-9	PTSD-SS	CFS	FSS	MRC Power	Grip Strength	Symptoms	Neuro Exam	Ocular Exam	6MWD	CPET	FIM	SGRQ	FIC	SDSS	SF-36	PSSS	SCSQ, SES, EPQ, Social Support, Threat to Life				
b1263	Psychic stability		+																														
b1266	Confidence									+																							
b130	Energy and drive functions											+		+	+												+						
b1302	Appetite											+																					
b134	Sleep function		+							+		+												+									
b144	Memory function																																
b152	Stress								+		+												+										
b152	Anxiety		+	+			+	+			+		+																				
b152	Depression			+	+	+		+		+	+	+															+						
b152	Panic																							+									
b152	Fear			+																				+									
b152	Concentrate		+							+		+		+																			
b1522	Anger		+																														
b1603	Control of Thoughts							+																									
b210	Seeing function																			+													
b255	Smell function																	+															
b265	Touch sensation																		+														
b280	Sensation of Pain																							+									
b440	Respiratory functions	+																															
b455	Exercise tolerance functions																				+	+		+	+								
b4552	Fatiguability													+	+									+									
b460	Sensation of respiratory system																	+						+									
b730	Muscle power functions															+	+		+														
s220	Structure of eyeball																			+													
d175	Solving Problems								+	+													+										
d177	Making decisions									+													+										
d310	Communication with – receiving – spoken language																						+										
d330	Speaking																																
d450	Walking																				+		+	+			+						
d460	Moving around in different locations																							+			+						
d510	Washing oneself																						+	+	+	+	+						
d520	Caring for body parts																						+			+	+						
d530	Toileting																						+			+							
d540	Dressing																						+	+		+							
d640	Doing housework																							+	+	+	+						
d710	Basic interpersonal interaction																						+			+	+						
d640	Formal Relationship																									+							
d760	Family Relationship																									+	+						
d770	Intimate Relationship																							+		+							
d845	Work and employment																							+		+							
d920	Recreation and Leisure																							+	+	+	+						
e310	Immediate family																											+					
e320	Friends																											+					

* Personal factors do not have second-level or third-level categories yet, “+” means that these ICF concepts were assessed by the item of the respective outcome measure [17].
